# Japanese encephalitis virus co-opts the ER-stress response protein GRP78 for viral infectivity

**DOI:** 10.1186/1743-422X-8-128

**Published:** 2011-03-20

**Authors:** Yi-Ping Wu, Chung-Ming Chang, Chun-Yu Hung, Meng-Chieh Tsai, Scott C Schuyler, Robert Yung-Liang Wang

**Affiliations:** 1 Department of Biomedical Sciences, Chang Gung University, TaoYuan, 33302, Taiwan; 2 Research Center for Emerging Viral Infections, Chang Gung University, TaoYuan, 33302, Taiwan

## Abstract

The serum-free medium from Japanese encephalitis virus (JEV) infected Baby Hamster Kidney-21 (BHK-21) cell cultures was analyzed by liquid chromatography tandem mass spectrometry (LC-MS) to identify host proteins that were secreted upon viral infection. Five proteins were identified, including the molecular chaperones Hsp90, GRP78, and Hsp70. The functional role of GRP78 in the JEV life cycle was then investigated. Co-migration of GRP78 with JEV particles in sucrose density gradients was observed and co-localization of viral E protein with GRP78 was detected by immunofluorescence analysis *in vivo*. Knockdown of GRP78 expression by siRNA did not effect viral RNA replication, but did impair mature viral production. Mature viruses that do not co-fractionate with GPR78 displayed a significant decrease in viral infectivity. Our results support the hypothesis that JEV co-opts host cell GPR78 for use in viral maturation and in subsequent cellular infections.

## Introduction

Japanese encephalitis virus (JEV) is a mosquito-borne flavivirus, a member of the family *Flaviviridae*, and causes serious viral encephalitis in humans [1,2]. JEV is a single-stranded positive-sense RNA genome of 11 kb nucleotides long, which contains a 5' cap structure but lacks a 3' polyadenylated tail [3,4]. This genomic RNA consists of a single open reading frame (ORF) flanked with two noncoding regions (NCRs) at the 5' and 3' ends [4]. The ORF is translated into a polyprotein precursor and subsequently processed into ten mature proteins by both host and viral proteases. The structural proteins are: the capsid (C), the premembrane (prM, which is further processed into pr and M), and the envelope (E) proteins; while there are seven nonstructural proteins; NS1, NS2A, NS2B, NS3, NS4A, NS4B, and NS5 [5]. The nonstructural proteins, together with cellular factors, form a viral replicase complex that directs the replication of the genomic RNA in the cytoplasm of the host cell, in association with perinuclear membranes [6,7]. During JEV assembly and release, it has been proposed that like other flaviviruses, immature virions are generally formed by the budding of a viral nucleocapsid into the endoplasmic reticulum (ER), where prM-E heterodimers are acquired. The mature virions are released into the extracellular compartment through the cellular secretory pathway [5,8].

Upon viral infection host cell protein expression is induced leading to the production of cytoplasmic proteins and secretory inflammatory cytokines. There is growing evidence that mature virus particles associate and/or contain host proteins once they are released from the host cell. These proteins may provide viruses with means to escape host immune defense or with a mechanism for its release as well as subsequent cell entry. For example, the differential expression pattern of secretion proteins from mock- and Dengus virus (DV)-infected HepG2 cells were identified and compared: eighty-six proteins have been identified among the secreted proteins of HepG2 cells [9]. In addition, proteomic analysis has revealed heat shock cognate protein 70 (HSC70) as part of the hepatitis C virus (HCV) viral particles. Down-regulation of HSC70 resulted in reduction of HCV virion release but not affecting HCV replication in cell culture system, suggesting that HSC70 modulates HCV infectivity [10].

The identification and functional analysis of secreted proteins from JEV-infected cells may reveal a role for host cell proteins in JEV pathogenesis. No global profile of secreted proteins from JEV-infected cells has yet been performed. To this end, we analyzed the effects of JEV infection on the profile of protein secretion of BHK-21 cells by developing a serum-free culture method in combination with LC-MS. We have identified 5 secreted proteins, including the molecular chaperones Hsp90, Hsp70, and GRP78. The role of GRP78 within the JEV life cycle was investigated. Our observations support the hypothesis that JEV co-opts GRP78 to play a role in viral infectivity.

## Results

### Proteomics analysis to identify secreted proteins upon JEV infection

**Figure 1:**
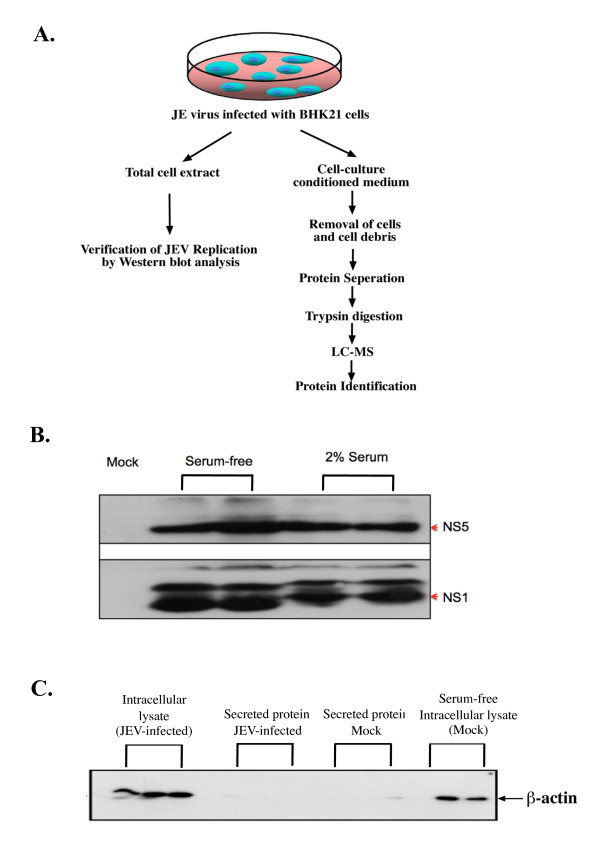
**An assay for the collection of secreted proteins from JEV-infected BHK-21 cells**. **A) **A flowchart outlining the protocol for the identification of JE virus replication from intracellular lysates and secreted proteins from the JEV-infected cells. **B) **Effect of serum deprivation on JEV replication. Cells were incubated with medium supplemented with 2% FBS or serum-free medium for 24 hours and viral proteins, NS1 and NS5, were assessed by Western blot analysis using anti-NS5/anti-NS1 polyclonal antibodies. Extracts from mock-infected cells serve as a negative control. Two independent replicates are shown for the serum-free and 2% serum conditions. **C) **Effect of serum deprivation on cell viability. β-actin was not detected in the secreted medium of mock- and JEV-infected BHK-21 cells. Three independent replicates are shown for the mock- and JEV-infected conditions.

JEV infection induces cellular protein secretion. In order to determine the optimal conditions to analyze secreted proteins during JEV infection, it was important to choose a period of secretion that allowed for maximal protein accumulation in the medium combined with minimal cell lysis or death. To this end, we devised an assay to search for JEV induced secreted proteins as shown in Figure 1A. Upon infection with JEV, the BHK-21 cells were cultured for two days in the presence of serum. The cells were then washed extensively to remove the proteins from fetal bovine serum present in the growth medium and cells were grown in serum-free media for an additional day before being harvested (Figure 1A). The cell extracts were isolated from serum and serum-free cultures and analyzed by Western blot to confirm viral replication under both conditions. The non-structural JE viral proteins, NS1 and NS5, were detected by anti-NS1 and anti-NS5 specific antibodies, respectively. The NS1 and NS5 expression levels were comparable under serum and serum-free culture conditions (Figure 1B), indicating the viral RNA replication was not affected by the removal of serum within the medium for one day. The collected serum-free secretion medium was separated from cells and cellular debris by centrifugation (8,500 × *g*, 10 mins). The highly abundant intracellular cytoskeleton protein, β-actin, was not detected in both serum-free and serum secretion media (Figure 1C).

**Figure 2:**
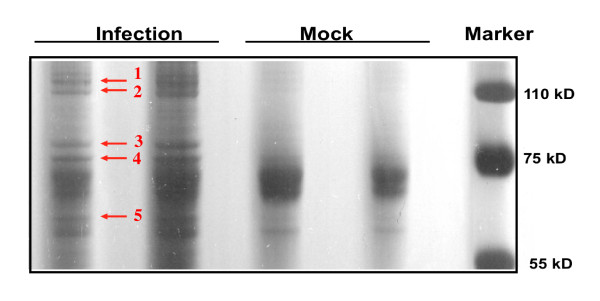
**Proteins identified by LC-MS in the secretion medium of JEV-infected BHK-21 cells**. Cell extracts were collected at three days after mock- and JEV-infection and subjected to one dimension SDS-PAGE analysis. A total amount of 10 μg of secreted proteins from mock- and JEV-infected BHK-21 cells was loaded per lane. The gel was stained with Silver nitrate. The identified proteins are shown as Table 1.

The secretion media was concentrated by ultrafiltration with a 10-kDa molecular weight cut-off, and the protein profile was analyzed by SDS-PAGE. Samples derived from mock-infected or infected cells were separated by 12% SDS-PAGE (Figure 2). There were 5 silver-stained bands that are unique in the JEV infected secretion medium compared to mock-infected medium (Figure 2). Gel bands were then subjected to in-gel trypsin digestion. The tryptic peptides were identified by liquid chromatography tandem mass spectrometry (LC-MS). The Mascot algorithm (Matrix Science, version 2.1) was employed for database searches. The most abundant proteins identified in each band were EF-2, Hsp90, GRP78, Hsp70, and cysteine ligase (Table 1). Since the induction of the unfolded protein response (UPR) accompanied by GRP78 up-regulation and cell death has been described for a number of viruses [11-15], we decide to investigate if there is a functional role for secreted GRP78 during the JEV life cycle.

**Table 1:** The protein identification of secretion medium upon JEV infection was identified by LC-MS.

Spot	NCBI number	Gene name	Protein name	Mass (Da)
1	NP_031933	Eef2	Elongation factor 2 [Mus musculus]	95,183

2	NP_032328.2	Hsp90ab1	Heat shock protein HSP 90-beta [Mus musculus]	83,150

3	NP_001156906	Hspa5	78 kDa glucose-regulated protein precursor [Mus musculus]	72,291

4	NP_034609.2	Hspala	Heat shock 70 kDa protein 1A [Mus musculus]	69,948

5	NP_080770	Ppcs	Phosphopantothenate--cysteine ligase [Mus musculus]	33,663

### GRP78 was present in JEV-infected secretion medium

**Figure 3:**
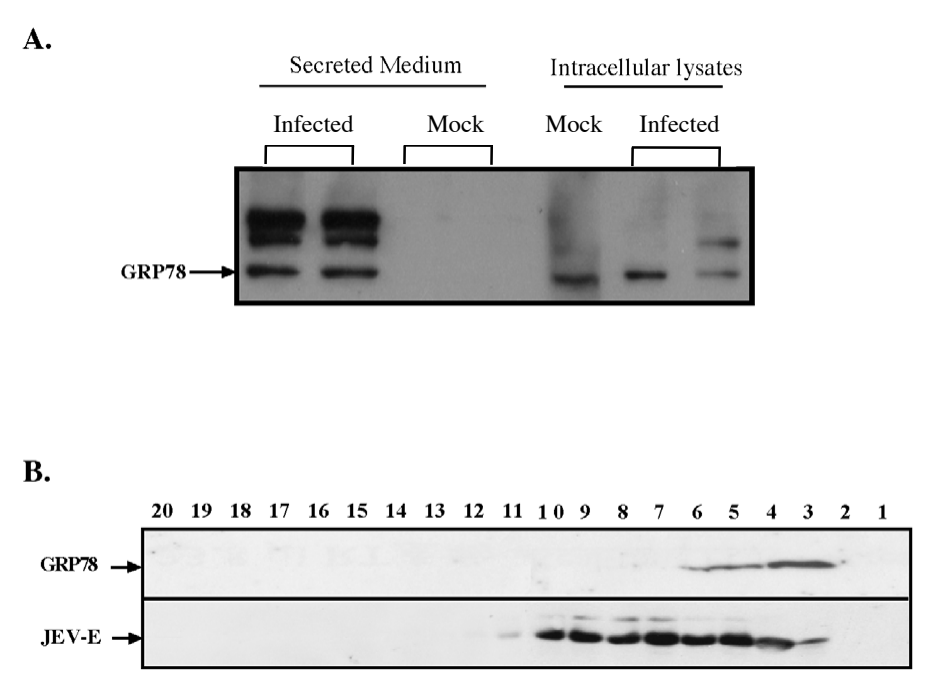
**GRP78 is released into the media upon JEV infection and partially co-fractionates with the JE virion**. **A) **Verification of GRP78 in the secretome from JEV-induced BHK-21 cells. Cell lysates or secretion medium were collected 3 days post-infection followed by SDS-PAGE for protein separation. The GRP78 was detected by anti-GRP78 specific antibody. Two independent replicates are shown for the mock- and JEV-infected conditions. **B) **Sucrose density gradient fraction of JE virion and GPR78. A volume of 40 μL of sample from each fraction was analyzed on SDS-PAGE followed by the detection of anti-JEV E protein and anti-GRP78 by Western blotting.

GRP78 is known to be resident primarily in the endoplasmic reticulum. It has been reported that GRP78 functions as a molecular chaperone involved in the folding process of nascent proteins, mostly through interaction with its peptide-binding domain [16-18]. GRP78 has also been demonstrated that serve as a co-receptor of viruses at the plasma membrane [19,20]. To validate and further characterize the secreted GRP78 in JEV-induced secretion medium, both intracellular extracts and secretion medium samples from mock-infected and JEV-infected cells were analyzed by Western blot. The detection of GRP78 from mock-infected and JEV-infected intracellular extracts were similar (Figure 3A), indicating that the GRP78 expression was not induced due to JE virus infection, which is in contrast with other reports [11,12,14,21]. Note that, the two upper bands shown in the JEV-infected secretion medium were probably GRP94 according to the report published by the Liberman group [22]. The GRP78 was detected only in JEV-infected secretion medium (Figure 3A), confirming the LC-MS result.

### GRP78 co-migrates with JE virus particles

Since the GRP78 was been detected only in the secretion medium from JEV-infected cells, we tested whether GRP78 was associated with JE virions. To this end, the secretion media collected from 3 dpi (days post infection) JEV-infected cells were subjected to 20%-60% continuous sucrose density gradient centrifugation. A volume of 0.5 mL fractions were collected and analyzed by Western blot. Co-migration of GRP78 with viral E protein was observed in fractions 3-6 (lane 3-6, Figure 3B). There were some fractions (fractions 7-10) where the viral E protein did not co-migrate with GRP78 (lane 7-10, Figure 3B). To further characterize the association of GRP78 with viral E protein, the secretion medium was treated with high-salt prior to sucrose density fractionation. No co-migration of GRP78 with viral E protein was observed (data not shown). These results indicate the association of GRP78 and viral E protein occur during viral particle release instead of during the centrifugation process.

### The co-localization of GPR78 with viral E protein in JEV-infected cells

**Figure 4:**
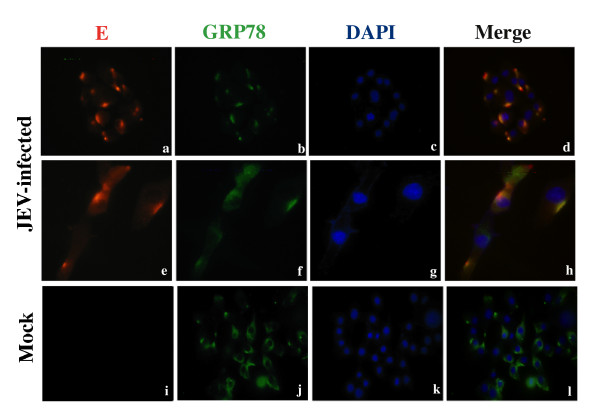
**The co-localization of GRP78 and JEV E protein in JEV-infected BHK-21 cells**. Mock- or JEV-infected BHK-21 cells were harvested at 3 days post-infection and prepared for immunofluorescence analysis stained with antibodies that detect GRP78 (green; b, f, j) and JEV-E protein (red; a, e, i).

It has been reported that GRP78 interacts and co-localizes with viral proteins (pp28) upon HCMV (human cytomegalovirus) infection. In HCMV infected cells, GRP78 was observed to relocate in the region of the endoplasmic reticulum near the periphery of the cells and the assembly compartment [23]. To investigate whether the GRP78 co-localizes with viral structural proteins within the cell, we performed immunofluorescent staining on GRP78 and viral E protein in mock- and JEV-infected cells, using antibodies specific for GRP78 and the viral E protein. GRP78 localized mainly in the cytoplasm of mock-infected cell. Co-localization of GRP78 with the viral E protein was observed (Figure 4), where GRP78 was detected in round, perinuclear structures resembling the cytoplasmic assembly compartment.

### Modulation of JE viral RNA release by GRP78

**Figure 5:**
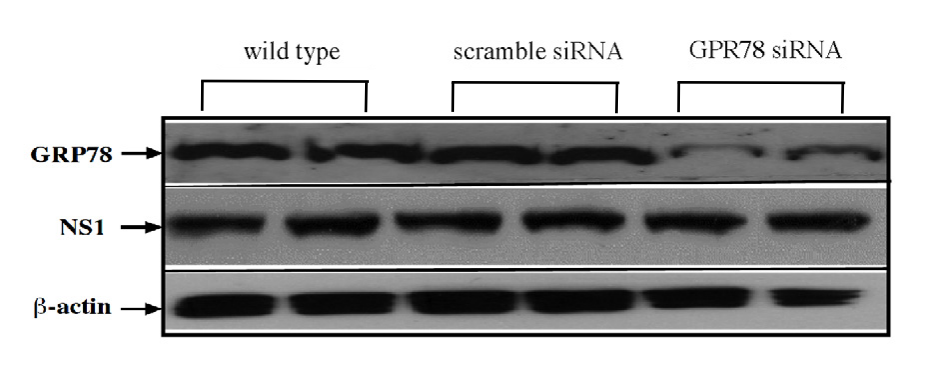
**Knocking-down the expression of GRP78 does not affect the replication of JE viral RNA**. BHK-21 cells were transfected with siRNA against GRP78 or an irrelevant siRNA (scramble siRNA) for 48 hours. The expression level of GRP78 was measured by Western blot analysis using polyclonal antibody specific to GRP78. Transfected cells were then infected with JEV at an MOI of 1. At 24 hour post-infection, the cell lysates were collected to measure the JEV replication using antibodies specific to NS1 and/or NS5. Two independent replicates are shown for the scramble and GRP78 siRNA conditions

**Figure 6:**
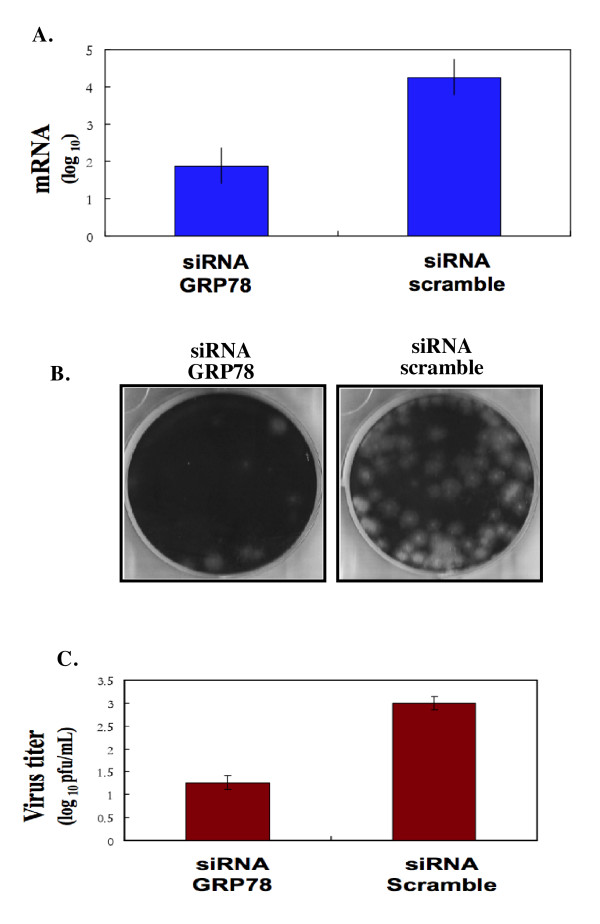
**Knocking-down the expression of GRP78 by siRNA decreases the yield of infectious JE virus production**. BHK-21 cells were transfected with siRNA against GRP78 or an irrelevant siRNA (negative control). The down-regulation of GRP78 was measured by Western blot analysis using antibody specific to GRP78 as shown in Figure 5. **A) **Transfected cell lysates were then infected with JEV at an MOI of 1. At 24 hours post-infection, the supernatants were collected to measure the amount of JE viral RNA production by RT real-time PCR as described in Material and Methods. The virus yield is expressed as a percentage of the yield obtained from cells transfected with irrelevant siRNA. **B) **Plaque formation by JE virus-particle collected from JEV-infected scramble siRNA treated cells or cells treated with siRNA against GRP78. **C) **Quantitative measurement of viral progeny produced from JEV-infected scramble siRNA treated cells or cells treated with siRNA against GRP78. The virus titer is defined as plaque-forming unit (PFU) per mL. Results are derived from three independent experiments.

Plus-stranded RNA virus infection has been observed to cause an increase in GRP78 expression for HCV [24], Enterovirus 71 (EV71) [25], and Dengue viruses [15]. In mammalian cells, the ER chaperone protein GRP78 functions as the principal sensor for the induction of the UPR and interacts with three modulators for the activation of cell apoptosis signal pathway. To evaluate the functional role of GRP78 in the JE viral RNA replication, the GRP78 was lowed by specific siRNA prior to JEV infection. GRP78 protein levels were reduced by GRP78-specific siRNA, but there was no observable effect on NS1 protein levels (Figure 5). The effect of reduced GRP78 expression on JE viral RNA release was also investigated. Two days after JEV infection in GRP78-specific siRNA treated cells, the extracellular JE viral RNA was decreased in comparison with cells treated with non-targeting sequences (Figure 6A). In contrast, no reduction was observed on the intracellular JE viral RNA (data not shown). The viral infectivity was also determined using a plaque-forming assay. An approximate 10-fold reduction in plaque formation was observed in GRP78-specific siRNA treated cells in comparison with cells treated with scramble siRNA (Figure 6B and 6C).

### JEV infectivity correlates with the presence of GPR78

**Figure 7:**
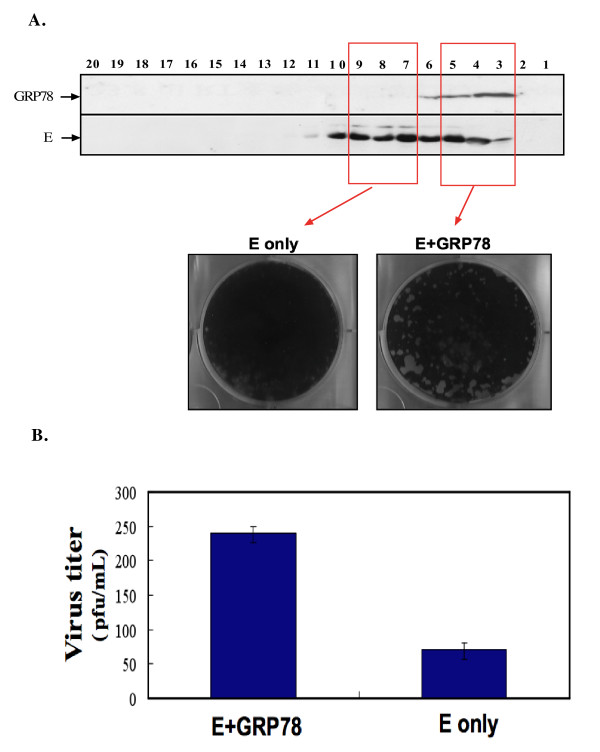
**Decrease in JEV infectivity in the absence of co-migrating GRP78**. **A) **Viral infectivity using plaque assay of JE virion-fractions associated without or with GRP78 was determined. **B) **Quantitative measurement of viral progeny produced from E+GRP78 and E only fractionates. The plaque assay results shown here are representatives of three independent experiments.

An effect of reducing GRP78 expression on JE viral RNA replication was not observed, but an effect on viral infectivity was found. We hypothesized that GRP78 may be co-opted by JEV to participate in viral infection. To test this hypothesis, the viral infectivity was measured using two distinct sucrose gradient fractions: those without (Figure 7A, also see Figure 3B, fractions 7-10) and those with co-migrating GRP78 (Figure 7A, also see Figure 3B, fractions 3-5). A decrease in viral infectivity was observed in the absence of co-migrating GRP78 (Figure 7B).

## Discussion

We analyzed the effects of JEV infection on the profile of proteins secreted by BHK-21 cells. Five secreted proteins were identified, including the molecular chaperones Hsp90, Hsp70, and GRP78. Co-migration of GRP78 with JEV particles in sucrose density gradients was observed and co-localization of viral E protein with GRP78 was detected by immunofluorescence *in vivo*. These observations suggest a physical interaction between JEV and GRP78. Knockdown of GRP78 expression by siRNA did not effect viral RNA replication, but did impair mature viral production. These data suggest that GRP78 does not modulate the intracellular replication levels of JEV, but instead is involved in the assembly or release steps of the viral life cycle. Mature viruses that do not co-fractionate with GPR78 displayed a significant decrease in viral infectivity. In combination, our results support the hypothesis that JEV co-opts host cell GPR78 for use in viral maturation and in subsequent cellular infections.

GRP78 is an endoplasmic reticulum (ER)-associated chaperone protein, a member of Hsp70 family. GRP78 is a major regulator of cell's unfolded-protein response (UPR), which is the cell's response to ER stress. In general, ER stress causes the sequestration of GRP78 and leads to the induction of a cascade of activation of proteins that can inhibit protein translation and assist protein refolding [26-29]. While GRP78 itself is protective against cell death [30], prolonged and extensive UPR and ER stress leads to apoptosis [26-29].

Induction of the UPR accompanied by GRP78 up-regulation and cell death has been described for a number of viruses, including bovine viral diarrhea virus [11], Tula virus [12], West Nile virus [13], Japanese encephalitis virus [14], and Dengue virus [15], the last four of which are in the flavivirus family. Infection by the hepatitis C virus (HCV), also a member of the family *Flaviviridae *and related to JEV, induces the GRP78 promoter and GRP78 mRNA levels are induced in cells expressing the HCV subgenomic replicon [24] or the HCV envelope [31,32]. Additionally, expression of the HCV structural proteins can induce GRP78 protein, ER stress, and CHOP-mediated apoptosis [33]. Recently, GRP78 has been shown to be up-regulated in DENV-infected cells and is necessary for DENV antigen production and/or accumulation. A similar report has also shown that GRP78 was up-regulated in the HCV-infected cells in an *in vivo *mouse model of HCV infection in association with ER stress and hepatocyte apoptosis [21]. Although there is strong evidence for the viral dependent induction of GRP78, the potential role of GRP78 in the viral life cycle is unclear. We have observed that knockdown of GRP78 expression by siRNA did not effect viral RNA replication, but did impair mature viral production, suggesting GRP78 is involved in the assembly or release steps of the JE viral life cycle.

Little is known about the exact mechanism of JEV (and other flavivirus) infectious particle assembly. For flaviviruses, some studies have identified a perinuclear structure, referred to as the cytoplasmic assembly compartment that is involved in the process [22]. Several viral proteins such as the structural protein E and prM and the nonstructural protein NS3 and NS1 have been reported to play an essential role in the process via an unknown mechanism [25]. It has been suggested that the virus directs specific viral and cellular proteins to the assembly compartment as needed for assembly compartment function. In our study, GRP78 co-localized with the viral E protein. Some studies have suggested that the formation of the assembly compartment may cause the condensation of other cytoplasmic structures [34,35]. These studies noted that the ER becomes located toward the periphery of the cell relative to the assembly compartment. The localization of GRP78 in nuclei and next to perinuclear structures may indicate co-localization with assembly compartments [23]. The intracellular co-localization of GRP78 with viral E protein upon JEV infection indicating that GRP78 was associated with the JE virus particle prior to release, which is similar to what was observed in this study. Our results, together with other reports, suggest that the GRP78-containing condensed ER structures is involved in the formation of the viral particle assembly compartment, especially in some flaviviruses.

JEV and/or other flaviviruses cell entry mechanisms are not well characterized. However, some links between viral infectivity and secreted proteins and, more specifically, chaperones have been described. The differential expression pattern of secreted proteins from mock- and Dengue virus (DV)-infected HepG2 cells were identified [9]. The up-regulation of signal peptide-containing secreted proteins in DV-infected HepG2 cells suggested that at least in part the secretion might be a result of the classical secretion pathway [36]. In addition, a proteomic analysis revealed that heat shock cognate protein 70 (HSC70) as part of the hepatitis C virus (HCV) viral particles. Down-regulation of HSC70 resulted in reduction of HCV virion release but not affecting HCV replication in cell culture system, suggesting that HSC70 modulates HCV infectivity [10].

Studies have revealed that following initial attachment to the cell surface JEV is recruited to the plasma membrane lipid raft (LR) prior to internalization of the particles. These studies suggested that flavivirus may use the LR as a platform to interact with additional host cell factors(s) required for efficient flaviviruses internalization. Because GRP78 does not contain transmembrane regions on the cell surface, we propose that GRP78 interacts with other factors to promote cell entry. Indeed, it has been reported that cell surface GRP78 interacts with diverse proteins, such as major histocompatibility complex class I molecules [20], the voltage-dependent anion channel [37], and the DnaJ-like protein MTJ-1 [38], all of which associate with LR in the plasma membrane [39-41]. Once JEV has attached to the cell surface, we speculate that it might utilize such GRP78-associated LR proteins for efficient cell surface attachment or internalization. In this study, we identified two other chaperones, HSP70 and HSP90 in the JEV-infected secretion medium in addition to GRP78 (Table 1). We hypothesize that both of the chaperones may interact with GRP78 to form the "chaperone-associated LR proteins" that facilitate more efficient cell surface attachment and/or internalization of the virus particle. Further studies are required for a full understanding of the cell association processes, especially receptor binding of JEV.

## Materials and methods

### Cell culture and Viruses

Baby Hamster Kidney-21 (BHK-21) cells were grown in RPMI 1640 medium (Gibco-Invitrogen, Carlsbad, CA, USA) supplemented with 5% fetal bovine serum (FBS) (Gibco-BRL, Carlsbad, CA, USA), 100 units penicillin (Gibco), 50 μg/mL streptomycin (Gibco-BRL, Carlsbad, CA, USA), and 24 mM sodium bicarbonate (Sigma, St. Louis, USA), and maintained at 37°C in an atmosphere of 5% CO_2_. The viral stocks were generated via γ-ray treatment of the Taiwan JEV NT109 strain, called RP-9 (provided by Dr. Ching-Len Liao, National Defense University, Taiwan). For infection of BHK-21 cells, we first replaced medium with serum-free RPMI-1640 medium for one hour, followed by infection with a multiplicity of infection (MOI) of 1 or 10. The JEV infected BHK-21 cells were then incubation for 2 days at 37°C in an atmosphere of 5% CO_2 _before being harvested for further experiments.

### RNA preparation and real-time PCR

RNA extraction was performed as described [42]. Briefly, total RNA was extracted with Trizol reagent (Invitrogen, Carlsbad, CA, USA) and viral RNA was extracted using QIAamp^® ^viral RNA mini kit (Qiagen, Hilden, Germany). JEV specific single-stranded cDNA was made from 2 μg of cytoplasmic RNA harvested from infected BHK-21 cells at 1 day post infection when infected at an MOI of 10. RNA was incubated with 10 μM of primer (5'-GCTAAGCATGTTCATCACTA-3'), and the reactions were carried out using the high capacity reverse transcription kit (Applied Biosystems, Carlsbad, California, USA) under conditions recommended by the manufacturer. The RT reaction was carried out at 37°C for 120 min, followed by PCR amplification of 2 μL aliquots of the TaqMan Fast Universal PCR Master Mix (Applied Biosystems, Carlsbad, California, USA) using an ABI 7500 Fast Real-Time PCR system (Applied Biosystems, Carlsbad, California, USA). The reactions were carried out under the following conditions: 95°C for 10 minutes, followed by 40 cycles of 95°C for 30 seconds and 60°C for 20 sec. The target sequences were amplified by using the following primer pairs and fluorogenic TaqMan probes: JEV RNA, forward (5'-GTTTTGGGAGCCTTACTTGT-3', corresponding to nt 3,642-3,662), reverse (5'-GCTAAGCATGTTCATCACTA-3', corresponding to nt 3,801-3,821), and probe (5'-6FAM-CATACCTCGCCAAATCA-MGBNFQ-3', corresponding to nt 3,689-3,705). Samples were run in 15 duplicates and a reaction without an aliquot of the RT reaction mixture was used to establish baseline fluorescence levels. Data were based on a threshold cycle (CT) in which the signal was higher than that of background. Quantitative analysis was dependent on the standard curve of standard sample Ct value and copy number, the RNA product of the standard sample were diluted from 10^11 ^to 10^6 ^of the copy number before RT.

### siRNA transfection assay

GRP78 siRNA were synthesized by Invitrogen with sequences of 5'-GUGCGUACGUAGCUAGC-3'. Scrambled siRNA was designed and synthesized by Invitrogen (medium GC of StealthTM RNAi negative control duplex, cat. No.12935). The siRNA transfection was conducted using Lipofectamine RNAiMAX (Invitrogen, Carlsbad, California), adding 10 μL Lipofectamine RNAiMAX and 10 μg of siRNA in 1 ml Opti-MEM (Invitrogen, Carlsbad, California), followed by incubation for 30 min at room temperature. The mixture was then added with 4 mL cell growth medium for 2 days, and the GRP78 protein was detected in siRNA transfected cells by Western blot using anti-GRP78 specific antibody (Abcam, Cambridge, MA).

### Western blotting

The protein samples of mock and JEV-infected BHK-21 cell lysates as well as secretion medium (collected from the supernatant of JEV-infected BHK-21 cells at 2 days post infection) were prepared by direct lysis of cell monolayers with 1x sample loading buffer (80 mM Tri-HCl pH 6.8, 2.0% sodium dodecyl sulfate (SDS), 10% glycerol, 0.1 M DTT, and 0.2% bromophenol blue). An equal amount of cell lysates was boiled for 5 min, separated by 12% SDS-PAGE under reducing conditions, and then electro-transferred to a methanol-activated polyvinylidene difluoride (PVDF) membrane (Bio-Rad Laboratories, Hercules, CA). The membrane was treated with 5% (wt/vol) nonfat dried milk in TBS-T buffer (20 mM Tris pH 8.8, 137 mM NaCl, and 0.1% Tween 20) at room temperature for 1 hour, followed by three 10-minute washes with TBS-T buffer. The membrane was then incubated in TBS-T buffer containing 0.5% nonfat dried milk at room temperature for 2 hours with a mouse anti-JEV NS1/NS5/E (1:1,000 dilution) (YaoHong Biotechnology Inc., New Taipei City, Taiwan), or rabbit anti-β-actin antiserum (1:10,000 dilution) (Sigma, St. Louis, USA). The primary antibody-decorated PVDF membrane was again washed three times with TBS-T buffer and incubated with an HRP-conjugated goat anti-mouse or anti-rabbit IgG (Sigma, St. Louis, USA), as appropriate, at a 1:5,000 dilution in TBS-T buffer containing 0.5% nonfat dried milk at room temperature for 1 hour. Following three 10-minute washes with TBS-T buffer the membrane was developed by ECL (Millipore, MA, USA).

### Viral plaque assay

BHK-21 cells were seeded in 6-well plates at 4 × 10^5 ^cells per well, followed by incubation overnight in RPMI 1640 medium containing 5% FBS to a form monolayer. The serial 10-fold dilutions of the sucrose gradient fractions or supernatant of JEV infected medium were prepared in serum-free RPMI medium before infection. After 1 day, the monolayer BHK-21 cells were incubated with serum-free RPMI 1640 medium for 1 hour and then the 0.5 mL of 10-fold dilutions and 0.5 mL of serum-free RPMI 1640 medium were added per monolayer BHK-21 for one hour. We prepared the 0.3% seaplaque agarose (Invitrogen, Carlsbad, CA) in 5% serum RPMI 1640 medium, adding 2 mL 0.3% seaplaque agarose per well after the monolayer cells washed with serum free RPMI 1640 medium. The 6 well TC plates were incubated at room temperature for 30 minutes to allow the 0.3% agarose overlay to solidify. The 6 well TC plates were then incubated at 37°C for 4 days. Finally, the cells were fixed with 2 mL of 10% formaldehyde, and kept for 30 minutes at room temperature (22-25°C), and the overlay of 0.3% agarose was removed. The monolayer cells were stained with crystal violet stain solution (0.5% crystal violet, 1.85% Formalin, 50% EtOH, 0.85% NaCl) (Sigma) for 2 minutes, and washed with ddH_2_O. The plaque-forming units (pfu/mL) was calculated with the virus titer formula, where virus titer equals the number of plaque × (1 mL/0.5 mL) × dilution factor.

### Sucrose density gradient analysis

The secretion medium of infected BHK-21 cells (2 days post-infection at MOI of 10) was centrifuged at 6,000 rpm for 20 minutes in 4°C to remove cell debris, and was concentrated with a concentration tube (Millipore, MA, USA) at 6,000 rpm for 20 minutes. The secretion and concentrated medium was layered onto a 20% to 60% sucrose linear gradient in HEPES buffer (20 mM HEPES, 0.5 mM EDTA, 50 mM KCl) and centrifuged at 40,000 rpm for 17 hours in 4°C. There were 10 fractions (1 mL/fraction) were harvested from the top of the sucrose gradients.

### Immunofluorescence and antisera

For immunofluorescent staining, cells were cultured on glass coverslips, rinsed with PBS twice, fixed with 4% paraformaldehyde in PBS for 30 min at room temperature, and then permeabilized with 0.1% (vol/vol) Triton X-100 in PBS for 30 min and incubated in 2% blocking buffer (Roche, Indianapolis, IN) for 1 hour. The cells were then incubated sequentially with primary antibodies: mouse anti-E protein (Yao-Hong Biotechnology Inc, New Taipei city, Taiwan); rabbit anti-GRP78 (Bioworld, Minnesota, USA) and secondary antibodies: (conjugated with Rodamine) and (conjugated with FITC). After immunostaining, coverslips were mounted on slides in gelvatol medium containing 4-6-diamidino-2-phenylindole (DAPI (Vector Laboratories, Inc., Burlingame, CA); 500 ng/mL in PBS). Images were acquired using a Zeiss confocal microscope (LSM 510) and processed with Adobe Photoshop software (Adobe, CA).

## Competing interests

The authors declare that they have no competing interests.

## Authors' contributions

RW conceived of the study, and drafted the manuscript. YPW & CMC carried out the virological and biochemical assays and drafted the manuscript. CYH & MCT participated in the design of the study. SCS participated in the design and the drafting of the manuscript. All authors read and approved the final manuscript.
