# Correction: Japanese encephalitis virus co-opts the ER-stress response protein GRP78 for viral infectivity

**DOI:** 10.1186/1743-422X-8-338

**Published:** 2011-07-05

**Authors:** Yi-Ping Wu, Chung-Ming Chang, Chun-Yu Hung, Meng-Chieh Tsai, Scott C Schuyler, Robert Yung-Liang Wang

**Affiliations:** 1 Department of Biomedical Sciences, Chang Gung University, TaoYuan, 33302, Taiwan; 2 Research Center for Emerging Viral Infections, Chang Gung University, TaoYuan, 33302, Taiwan

## Correction

While reviewing the proof of our article [1] after it was published on-line in its provisional form, we noticed two incidences where sentences in our article share a high level of similarity with two previously published papers. The first incidence is in the first paragraph of the results section where four sentences in our article share similarity to sentences in the results section of a previously published article [2]:

"In order to determine the optimal conditions to analyze secreted proteins during JEV infection, it was important to choose a period of secretion that allowed for maximal protein accumulation in the medium combined with minimal cell lysis or death. To this end, we devised an assay to search for JEV induced secreted proteins as shown in Figure one A. Upon infection with JEV, the BHK-21 cells were cultured for two days in the presence of serum. The cells were then washed extensively to remove the proteins from fetal bovine serum present in the growth medium and cells were grown in serum-free media for an additional day before being harvested (Figure one A)."

"The secretion media was concentrated by ultrafiltration with a 10-kDa molecular weight cut-off, and the protein profile was analyzed by SDS-PAGE."

The second incidence was in the second and third paragraph of the discussion section where six sentences in our article share similarity to sentences in the introduction of a previously published article [3]:

"While GRP78 itself is protective against cell death, prolonged and extensive UPR and ER stress leads to apoptosis.

Induction of the UPR accompanied by GRP78 up-regulation and cell death has been described for a number of viruses, including bovine viral diarrhea virus, Tula virus, West Nile virus, Japanese encephalitis virus, and Dengue virus, the last four of which are in the flavivirus family. Infection by the hepatitis C virus (HCV), also a member of the family Flaviviridae and related to JEV, induces the GRP78 promoter and GRP78 mRNA levels are induced in cells expressing the HCV subgenomic replicon or the HCV envelope. Additionally, expression of the HCV structural proteins can induce GRP78 protein, ER stress, and CHOP-mediated apoptosis. Recently, GRP78 has been shown to be up-regulated in DENV-infected cells and is necessary for DENV antigen production and/or accumulation. A similar report has also shown that GRP78 was up-regulated in the HCV-infected cells in an in vivo mouse model of HCV infection in association with ER stress and hepatocyte apoptosis."

Upon making this discovery, we immediately contacted the Editor-in-Chief to make the journal aware of the issue. Subsequently, we conducted our own investigation and provided a full report to the publisher, who performed an independent investigation.

We would like to sincerely apologize to our colleagues in the field for this mistake, whose work was both inappropriately utilized and not properly referenced. We would also like to apologize to the reviewers of our manuscript and the journal Editors. We are grateful for the opportunity to acknowledge and correct this mistake.
